# Health and use of health services of people who are homeless and at risk of homelessness who receive free primary health care in Dublin

**DOI:** 10.1186/s12913-015-0716-4

**Published:** 2015-02-12

**Authors:** Claire Keogh, Kirsty K O’Brien, Anthony Hoban, Austin O’Carroll, Tom Fahey

**Affiliations:** HRB Centre for Primary Care Research, Department of General Practice, Royal College of Surgeons in Ireland, 123 St Stephen’s Green, Dublin 2, Ireland; Safetynet, Coolmine House, 19 Lord Edward Street, Dublin 2, Ireland

## Abstract

**Background:**

Homeless populations experience poorer physical and mental health, and more barriers to accessing adequate healthcare. This study investigates the health of this population, following the provision of a free to access primary care service for homeless people in Dublin (Safetynet). The health of this group will be compared to previous studies on homelessness conducted in Dublin prior to the establishment of this service (in 1997 and 2005).

**Methods:**

Participants were recruited through Safetynet clinics. A 133-item questionnaire was administered to determine participants’ physical and mental well-being, use of health services and healthcare needs. Prescription data was extracted from participants’ electronic health records.

**Results:**

A total of 105 participants were recruited. The majority were < 45 years of age (69%), male (75%), single (52%), Irish (74%) and had children (52%). Multimorbidity was common; with 5.3 ± 2.7 (mean ± SD) physical conditions reported per person. A large proportion of participants had at some point received a formal diagnosis of a mental health condition (70%; 73/105), including depression (50%; 52/105), addiction disorder (39%), anxiety (36%; 38/105), schizophrenia (13%; 14/105) and bipolar disorder (6%; 6/105). With regards to illicit drug use, 60% (63/105) of participants reported ever using drugs, while 33% (35/105) reported being active drug users. Based on AUDIT C criteria, 53% had an alcohol problem. Compared to previous studies, participants reported more positive ratings of health (70% vs. 57% in 1997 and 46% in 2005). The proportion of participants on one or more prescription medication was higher than in previous studies (81% vs. 32% in 1997 and 49% in 2005) and there was a decrease in attendance at outpatients departments (17% vs. 27% in 2005) and a trend towards a decrease in attendance at Accident and Emergency departments (A & E) (29% vs. 37% in 2005).

**Conclusions:**

This vulnerable population has many physical and mental health problems. Use of drugs, alcohol and smoking is common. Following the establishment of Safetynet, self-reported health was rated more positively, there was also a decrease in the use of A & E and outpatient services and an increase in prescription medicines.

**Electronic supplementary material:**

The online version of this article (doi:10.1186/s12913-015-0716-4) contains supplementary material, which is available to authorized users.

## Background

Homelessness is associated with higher rates of mortality, morbidity, poor mental health, alcohol and drug use and other risky health behaviours relative to the general population, making them a particularly vulnerable group. [1-5]. Despite the volume of healthcare needs, homeless populations face a number of barriers to receipt of appropriate services, including lack of engagement with services and continuity of care; lack of medication adherence and a lack of coordination between healthcare services, negative prior experiences, perceived prejudice towards homeless people, difficulty affording prescribed medication and medication storage [6,7].

General practice has been identified as an important setting to meet these needs and to provide an opportunity for early intervention [8-10]. This has been shown to improve the health of the homeless and reduce hospital admissions, resulting in reduced overall cost to the healthcare system [9].

In Ireland, in 2011, there were approximately 3,800 homeless people with the majority (2,375) living in Dublin [1]. In Ireland the majority of people pay to visit a GP and for their medication; around a third of the population is entitled to a means tested free healthcare scheme (GMS scheme). Those on the scheme have free access to GPs and prescribed medication. However, it is known from previous studies on the health of homeless people in Dublin that only around 55% of the homeless population have joined the scheme [2,11]. Safetynet was established in 2007 and provides homeless people, and those at risk of homelessness, with free access to primary care workers including GPs, nurses and drug workers regardless of GMS status. The clinics are all based in homeless shelters and foodhalls to allow easy access. It is supported by the Health Service Executive (HSE) and consists of 14 clinics across Ireland, although these are predominately based in Dublin. The clinics use electronic patient record sharing, which allows patients to be treated in any of the clinics participating in the network. The multidisciplinary team available within the Safetynet network and the increased flexibility of this approach should allow increased access to healthcare for homeless patients and potentially lead to better health of this population.

The aim of the current study was to investigate the health and use of healthcare services of a homeless population in Dublin who access the Safetynet services. This will allow us to characterise the population who access this service and to compare the health of the Safetynet population to studies completed prior to its establishment [2,11].

## Methods

Ethical approval for this study was granted by the Royal College of Surgeons Research Ethics Committee.

### Study design and setting

This observational cross sectional study was conducted according to the STROBE guidelines [12]. Recruitment took place during an eight week period in summer 2011, using a convenience sampling method. Prescription information was collected from the electronic health record retrospectively.

### Participants

Two medical student researchers recruited participants from four of the Safetynet health clinics across Dublin city centre. Recruitment continued until saturation.

### Participant recruitment

Each of the participating centres requested a gate-keeping mechanism, whereby patients were first informed of the study by a member of Safetynet staff. Interested participants were then introduced to the researchers, who were on site. Participants provided informed consent prior to participation. Each item on the consent form was read aloud to the participant by the researcher and participants could choose to participate in certain elements of the study, including: (i) collection of prescribing information from the electronic Safetynet patient record at baseline and (ii) at 12 month follow-up.

### Questionnaire

The questionnaire used in the current study modified and extended that used in two previous studies investigating the health of the homeless in Dublin in 1997 [11] and 2005 [2]. The questionnaire consisted of 133 items that assessed patients’ reasons for homelessness, health and well-being, risky health behaviours and use of health services. Participants were asked to report if they ‘currently’ or ‘ever’ experienced a list of mental and physical health problems. In addition, the current study included a number of standard questionnaires used to assess patients’ mental health and quality of life. These included the AUDIT C to measure problem drinking; the GAD-7 to measure generalised anxiety; PHQ-9 to measure depression; and the SF-12 to measure quality of life. The AUDIT C is scored on a scale of 0–12 with 0 representing no alcohol use, while a score of ≥4 in men or ≥3 in women indicates alcohol misuse [13]. For the PHQ-9, ≥10 indicates the possibility of clinically significant depression, while ≥15 indicates severe depression. For the GAD-7, ≥ 10 indicates the possibility of clinical anxiety, while a score of ≥15 suggests severe anxiety. The SF-12 measures eight health domains including physical functioning, role-physical, bodily pain, general health (physical component summary; PCS) and vitality, social functioning, role emotional and mental health (mental component summary; MCS). According to this norm based scoring system, an average healthy adult would score 50, if a group scores below 47 then the health of the group is lower than average. The questionnaire took approximately 45 minutes to complete, with a researcher (AH or MC) asking the patients the questions and writing down their verbal responses.

### Prescribed and Over the Counter (OTC) medications

**Figure 1:**
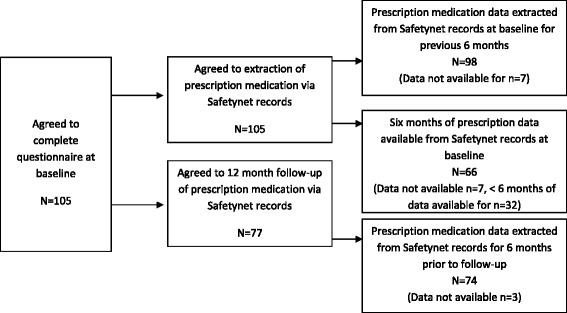
**Flow of participants throughout the study.**

Medication information was collected from three sources: self-reported prescribed and over-the-counter medications; Six months worth of data from the Safetynet electronic patient health record and non-Safetynet GPs (where relevant). Data from the electronic patient record was collected for the six month period prior to the interview date and from 6–12 months after the interview date (Figure 1).

### Previous studies

Two previous studies, conducted in 1997 [11] and 2005 [2] used a shorter version of the questionnaire used in this study. In the 1997 study, they recruited participants from hostels, B & Bs, food halls and soup runs that cater for homeless people, while the 2005 study recruited from homeless hostels and B & Bs only.

### Plan of analysis

This study provides a descriptive analysis of the health and healthcare needs of this population. The prevalence of health issues will be reported. Descriptive comparisons will be made between the current study and those studies conducted in Dublin in 1997 and 2005 [2,11].

## Results

### Participants

The flow of participants through the study is presented in Figure 1. A total of 105 participants were recruited from the four clinics during the baseline recruitment period.

### Demographics of the Safetynet population

Participant demographics for the Safetynet cohort at baseline are presented in Additional file 1: Table S1. The majority of participants were less than 45 years of age (69%; 72/105), male (75%; 79/105), single (52%; 55/105), Irish (74%; 78/105) and had children (52%; 54/104). This was largely in keeping with a homeless census which was conducted Nationally in 2011 [1]. The majority of participants had been in their current accommodation for between 1–12 months (51%; 54/105) and were living in a hostel (51%; 53/104). More than half of participants reported that they were satisfied or very satisfied with their current residence (65%; 68/105). Drug and alcohol addiction (28%; 29/103), financial reasons (24%; 25/103) and family related problems (23%; 24/103) were commonly cited as the primary reason for homelessness. However, the reason for homelessness was often multifaceted, with participants generally citing more than one reason.

### Health and morbidity of the Safetynet population

Details on health and morbidity are presented in Additional file 2: Table S2. The majority of participants rated their physical health (70%) and mental health (75%) as good to excellent. Despite this, 97% of the population reported having at least one current physical or mental health condition. The average number of physical conditions reported per person was 5.3 ± 2.7 (range 1–11). Most of the participants were experiencing multimorbidities, with 84% (88/105) reporting currently having two or more mental or physical conditions.

The most common physical health problems included: skin (52%, 55/105); dental (48%; 50/105); eye (31%; 32/105); joint (23%; 24/105) and asthma (21%; 22/105). Of the blood-borne diseases, the most commonly reported disease was hepatitis C (23%; 24/104).

A large proportion of participants had at some point received a formal diagnosis of a mental health condition (70%; 73/105), including depression (50%; 52/105), anxiety (36%; 38/105), schizophrenia (13%; 14/105) and bipolar disorder (6%; 6/105). More than a third of the population had been formally diagnosed with an addiction disorder (39%; 41/105), that was predominantly related to drugs (72%, 28/39), with smaller numbers diagnosed with an addiction to alcohol (28%, 11/39) and gambling (3%, 1/39). A total of 18% (19/105) of the population reported feeling suicidal in the previous 6 months, while 9% (9/105) reported a suicide attempt during the same period.

The PHQ9 indicated that 20% (21/105) met the criteria for major depression (moderately severe), while a further 21% (22/105) were at high risk of depression. The results from the GAD-7 indicated that 17% (18/105) met the criteria for clinical anxiety (severe anxiety), while a further 30% (31/105) were at risk of anxiety problems. There was a highly significant association between those who met the PHQ9 criteria for major depression and those who met the GAD7 criteria for clinical anxiety, with 78% (14/18) who had a GAD7 score ≥15 criteria also having clinical depression based on PHQ9 (χ^2^ = 45.3, df 1, p = 1.7 × 10^−011^).

The results from the SF-12 indicated that the Safetynet population reported below average mental health with a mean MCS score of 43.5 ± 12.5 (range 6.7-66.2). The mean PCS score was 48.1 ± 10.8 (range 14.0-70.0), which is just above the cut-off for average health (average ≤47).

### Risky health behaviours

The prevalence of risky health behaviours is presented in Additional file 3: Table S3. A total of 82% (86/105) of the population were current smokers, of which the average age of first smoking was 15.2 ± 7.4 years (range 5–63 years) and they had smoked on average for 22.4 ± 11.5 years (range 1–62 years.). Of current smokers, 95% reported smoking every day for the last 3 months, with the majority smoking 10–20 cigarettes per day. Over half of the population (58%; 61/105) reported currently drinking alcohol. According to the AUDIT-C criteria, 53% of the total population (92% of current drinkers) met the criteria for problem drinking. The average age of first alcohol consumption was 16 ± 4.2 years (range 6–35 years).

With regards to illicit drug use, 60% (63/105) of participants reported ever using drugs, while 33% (35/105) reported being active drug users. The most commonly used drugs in the previous 90 days were cannabis (n = 27), heroin (n = 25) and benzodiazepines (not prescribed) (n = 19). A total of 44% (46/105) of the population reported ever injecting drugs, with 22% (23/105) injecting drugs within the past 3 months. Of those that have ever injected drugs, over half had re-used their own needle/syringe (56%; 26/46), with high proportions also reporting using a filter, spoon or flush used (43%; 20/46) or injected using a needle or syringe (39%; 18/46) used by somebody else.

Of the participants who had sex with someone other than their regular partner (n = 10), 60% (6/10) did not use a condom on every occasion. One fifth of participants (21/104) reported that they had ever paid for sex, of which 29% (6/21) did not use a condom on every occasion.

### Prescription medication

Based on self report, the majority of participants were receiving prescribed medications (81%; 85/105). Prescription data was extracted from the Safetynet electronic patient record for all participants who consented (n = 98, Figure 1) and had been using the service for a minimum of six months (n = 66). For participants who also attended a GP outside of Safetynet (n = 52/105), data was received for 8 participants. At baseline, 64% (42/66) were prescribed at least one medication during the last six months and the majority of these participants were experiencing polypharmacy (4 or more medications [14]), 74%; 31/42, Additional file 4: Table S4).

**Figure 2:**
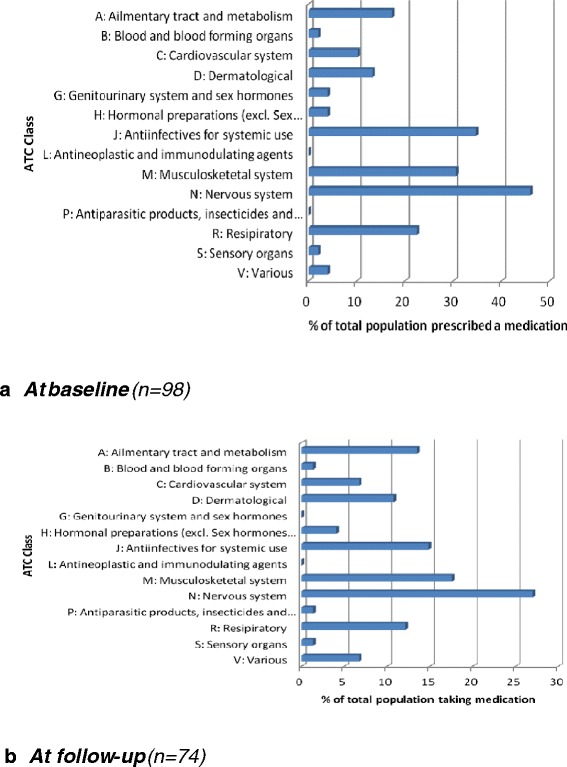
**Medication prescribed by ATC class (count represents the number of participants receiving at least one prescription of a drug in the ATC class during the previous six months). a)** At baseline (n=98), **b)** At 12 month follow-up (n=74).

Prescribing data was categorised according to WHO Anatomical Therapeutic Chemical Classification System (ATC) (Figure 2). Although many of the drugs were on repeat prescription, the count here represents the number of participants receiving at least one prescription of a drug in the ATC class during the previous six months (n = 98). The most commonly prescribed drugs were for the nervous system (ATC N class), 46% (45/98) of participants had been prescribed an N class drug in the previous 6 months. These included analgesics (N02) (n = 22/98), most commonly, paracetamol (N02BE01; 15/22). Drugs used in addiction (N07B)(14/98), most commonly methadone (9/14). Psycholeptics (N05) (n = 12/98), most commonly zopiclone (6/12) and antidepressants (N06) (n = 9/98).

Anti-infectives for systemic use (ATC J Class) were the second most commonly prescribed drugs, with 35% (34/98) receiving a systemic antibiotic (J01). This was followed by drugs acting on the musculoskeletal system (ATC M class), specifically anti-inflammatory and anti-rheumatic drugs (M01) (29%; 28/98), most often ibuprofen (n = 22/28). Drugs acting on the respiratory system, such as those for obstructive airways diseases (R03), were prescribed to 13% (13/98).

At 12 month follow-up, the prevalence of prescribing was lower than at baseline at 43% (32/74). The pattern of prescribing was largely similar to that reported at baseline (Additional file 4: Table S4, Figure 2).

### Use of health services

Details on the use of health services are provided in Additional file 5: Table S5. Of the 41% (43/105) of participants who did not have a medical card, the majority (44%; 18/41) reported they had applied for one but were waiting for a response.

More participants reported using primary care services (including Safetynet) in the previous six months than other medical services. This included the GP (82%; 86/105) and Safetynet nurses (44%; 46/105). On average, participants reported that they visited the GP 6.5 ± 6.3 times (range 1–30) in the previous six months. Half of the participants reported attending a non Safetynet GP (52/105), but the majority (82%; 84/103) used Safetynet GPs more often. The most common reason for their last visit to a Safetynet GP was for methadone (n = 11/95), followed by wound care or dressings (n = 7/95), pain (n = 7/95) or respiratory tract complaints (n = 7/95). The majority of participants reported that they would have gone nowhere else (35%; 33/93), to another GP (26%; 24/93) or to accident and emergency (A & E) department (23%; 21/93) on their last visit, if a Safetynet GP were not available.

A total of 29% (30/105) of participants attended A & E and 17% (18/105) attended the outpatient department in the previous six months. These were generally for reasons that could not necessarily be addressed in primary care (e.g. falls, fractures, pneumonia and assault).

Over a fifth (21%; 22/104) of the population (86% of current intravenous drug users) reporting the use of needle exchange services in the previous 6 months.

Over half of those with a current mental health diagnosis reported using mental health services (59%, 35/59). A small number of participants (3%; 3/104) reported being on waiting lists for counselling, psychiatric care or supervised detoxification. Almost a third of the population (29%; 30/105) indicated that they were currently receiving methadone treatment, with the average duration of treatment being 3.3 ± 4.5 years (range 1 week-17 years). Four percent of the participants were on a waiting list for methadone treatment for up to 12 weeks.

### Comparisons with previous homeless populations

Similarities were observed across the three populations in terms of basic demographics (Additional file 1: Table S1). However there was an increase in the proportion of participants that were from mainland Europe in the current study (14% vs. 5% in 1997 and 1% in 2005), including Romania (n = 10) and Poland (n = 5). The majority of participants in all three studies were staying in hostels. However the current study also included people owning/renting their own accommodation due to the inclusion of those “at risk of homeless” and this accounted for more than quarter (26%) of the study population. Fewer participants in the current study reported family problems (23% vs. 32% in 1997 and 37% in 2005) and more reported financial reasons (24% vs. 20% in 1997 and 13% in 2005) as the primary reason for homelessness.

Differences emerged in terms of the health and morbidity of participants across the three studies (Additional file 2: Table S2). A larger proportion of participants in the Safetynet population rated their health as good to excellent (70% vs. 57% in 1997 and 46% in 2005), despite the increase in prevalence of mental and physical health conditions. The prevalence of hypertension, respiratory disease, skin problems and peptic ulcer disease were all higher in this study, while arthritis and hepatitis C was lower than in previous studies. Rates of schizophrenia, depression and anxiety were similar to 2005.

The rate of current smokers remained high (Additional file 3: Table S3). However, there was a decrease in the prevalence of heavy smoking (defined as >30 cigarettes per day) since 1997. Current drug use had increased since 2005, although the percentage of participants who had ever injected drugs was similar to 2005 rates (48% in 2005, 44% in this study). Based on recommended drinking limits (≤14 units for a woman and ≤21 units for a man per week), alcohol consumption had not increased (26% vs. 25% in 1997).

The proportion of participants with a medical card (59% vs. 55%) was similar to 2005. However, a difference emerged in the most common reason for not having one; from difficulties getting a GP to sign forms (2005) to having applied for one but awaiting a response (current study). The proportion of people who had attended a GP in the previous six months had increased since the 2005 study (82% vs. 62%, χ^2^ = 14.3, df = 1, p = 0.0002), whilst a decrease for the same period was observed in the numbers attending the outpatients department (17% vs. 27%, χ^2^ = 4.1, df = 1, p = 0.0439). Although not statistically significant there was a trend towards a decrease in attendance at A & E in 2011 compared to 2005 (29% vs. 37%, χ^2^ = 2.5, df = 1, p = 0.1131) and there was also a small increase in the number of participants who were hospital inpatients in the previous 6 months (19% vs. 22%, χ^2^ = 0.245, df = 1, p = 0.6208).

## Discussion

### Summary of main findings

The majority of participants were young (under 45 yrs), male and Irish. The most common reasons cited for homelessness included drug and alcohol addiction (28%), financial reasons (24%) and family reasons (23%). There was a difference between this study and previous studies in terms of reasons for homelessness (more participants reported financial reasons as their primary reason for homelessness) and in terms of the ethnic origin of participants (more mainland Europeans); both these factors are a reflection of Ireland in 2011. Ireland was in economic recession during 2011, with many people losing jobs coupled with a tightening of the social welfare system. The enlargement of the European Union in 2007, led to an increase in the number of mainland Europeans living in Ireland.

Almost three quarters of participants rated their physical and mental health as good to excellent; this represented an increase in self rated health compared to previous studies. The vast majority of participants (84%) were experiencing multimorbidity. The most common physical complaints were chest infections, skin, dental, eye, joint, and asthma problems. Almost a quarter of participants (23%) had hepatitis C. The most common prescription medications were ATC class N (nervous system) medications (e.g. paracetamol), as well as class J (anti-infectives for systemic use) medications (e.g. systemic antibiotics). A large proportion of participants (70%) reported a mental health condition, with depression (50%), addiction disorder (39%), anxiety (36%) and schizophrenia (13%) being the most common conditions reported. Compared to the previous homeless studies, an increase was seen in the numbers of participants reporting physical or mental health problems and the numbers of participants receiving a prescription.

Risky health behaviours were very common in this study with 82% currently smoking, 58% currently drinking and a third were current drug users. Compared to previous studies, Safetynet participants reported higher levels of current illicit drug use.

Participants often used primary care services, with participants visiting their GP on average 6.5 times in the previous 6 months. Almost a third of participants were on methadone and 29% reported attending an A & E department in the previous 6 months. Compared to previous homeless studies, there was an increase in attending a GP, a decrease in those attending outpatient departments and a trend towards a decrease in attendance at A & E.

### In context of other studies

A number of studies have shown an increased rate of infectious diseases in the homeless population compared to the general population and this was reviewed in 2014 by Fazel et al. [15]. This review only included homeless populations from high income countries. The prevalence reported for tuberculosis, hepatitis C and HIV are similar to our study; however the participants in this study reported a lower rate of hepatitis B compared to the review (17-30% vs. 3%). This may be due to a third of the population having received a hepatitis B vaccination. For non-communicable diseases, the prevalence of diabetes is in keeping with what has previously been reported for homeless populations in Ireland, France and Portugal [15], but rates of myocardial infarction, stroke and hypertension are lower than reported in the USA and Canada. Hypertension was reported by 22% of participants in this study (compared to 50% in USA and 35% in Canada), however it is not clear if this is due to underdiagnosis rather than a true lower prevalence. The incidence in the general population of deep vein thrombosis is around 1 in 1000 [16], however, a high proportion of participants in this study reported deep vein thrombosis (DVTs) (19%). Although not reported previously to be specifically linked to homelessness, others have reported a link between intravenous drug use and DVTs [17]. High rates of multimorbidity has been reported here (84%) and by other studies ([18], 66%).

Around 40-50% of our participants had depression (either self reported or based on PHQ-9 scores), and 39% reported an addiction disorder; this is in keeping with a meta-analysis on the prevalence of mental disorders among homeless people in western countries [3], which found 0-49% had depression and 5-58% had alcohol or drug problems. The prevalence of self reported schizophrenia in this study was the same to that reported in a large Danish cohort study (13% vs. 13%).

Our participants had a high rate of smoking, this has been reported in many other homeless populations [3,19] and is thought to contribute to the high levels of morbidity and mortality in this population.

### Strengths and limitations

This is the first study to look at the health of the homeless in Dublin since free access to primary care services was made available for this population, through Safetynet.

The recruitment strategy in this study differed to the previous studies in 1997 and 2005 [2,11] and this may have led to selection bias. In this study, participants were recruited from within Safetynet Clinics, while the previous studies recruited directly from shelters, B & Bs, food halls and soup runs. However, it is worth noting that Safetynet clinics are located in homeless shelters and food halls, allowing easy access for anyone attending these facilities. This could mean that the current sample may not be representative of all homeless people living in Dublin, but rather those that are motivated to attend for healthcare. Differences also existed across the studies in terms of their definition of homelessness, with the current study including participants ‘at risk of homelessness’. Although the inclusion of those ‘at risk of homelessness’ may have potentially biased our results, a subgroup analysis was done to compare the difference between our whole cohort (n = 105) and the cohort without this group (n = 77) in terms of quality of life, health outcomes, health behaviours and health care utilisation and no significant difference was found between the groups (p > 0.05). The sample size is small compared to previous studies, however, the recruitment process continued until no new participants could be found in each of the four clinics that permitted access for recruitment.

We extracted participants’ prescription data from the Safetynet electronic health record, however, 50% (52/105) of participants reported also attending an additional non-Safetynet GP. Although we requested this information from these GPs, data was only received for 8 participants. As such, it was not possible to draw unbiased conclusions on the appropriateness of prescribing.

### Clinical implications

The results indicate that the Safetynet population have more healthcare needs than the general population, in terms of mental and physical health problems, risky health behaviours and greater use of health services. This makes them a vulnerable population. However, their health needs are similar to that of other homeless cohorts. Since Safetynet was established, there has been an increase in use of GP services, an increase in the diagnosis of health problems and an increase in the rate of prescribing in a homeless population in Dublin.

As almost a quarter of participants reported that they would have attended an A & E department for their last healthcare visit, had a Safetynet GP not been available, the implementation of a free primary health care service may have reduced the burden on A & E services.

Nevertheless, participants in the current study reported high levels of problem health behaviours which will likely result in future adverse outcomes [20]. Furthermore, high rates of suicidal thoughts and suicide attempts were reported in this population. This provides a further challenge to Safetynet healthcare workers and suggests one area for future development of healthcare interventions.

## Conclusions

This study highlights the challenges to the increasing and complex health and healthcare needs of a homeless population in Dublin, relative to the previous homeless studies.

## Additional files

Additional file 1: Table S1. Demographics of participants in current study at baseline compared with previous Irish studies. Additional file 2: Table S2. Health and morbidity of participants in current study at baseline compared with previous Irish studies. Additional file 3: Table S3. Health behaviours of participants in current study at baseline compared with previous Irish studies. Additional file 4: Table S4. Medications information. Additional file 5: Table S5. Use of health services by participants in current study compared with previous Irish studies.
