# Supplementary material for: Health and use of health services of people who are homeless and at risk of homelessness who receive free primary health care in Dublin
Source: BMC Health Serv Res. 2015 Feb 12;15:58. doi: 10.1186/s12913-015-0716-4 (PMC4343065; doi:10.1186/s12913-015-0716-4)
Supplement: Additional file 1: Table S1. — Demographics of participants in current study at baseline compared with previous Irish studies. [file 12913_2015_716_MOESM1_ESM.docx]

**Additional file 1: Table S1: Demographics of participants in current study at baseline compared with previous Irish studies**

| Demographic | Holohan et al 1997  (n=502)  %(n) | O’Carroll et al 2005 (n=356)  %(n) | Current Study 2011 baseline data (n=105)  %(n) |
| --- | --- | --- | --- |
| Age |  |  |  |
| Under 45 years of age | 65% (319/493) | 81% (289/355) | 69% (72/105) |
| Over 65 | 8% (40/493) | 1% (5/355) | 4% (4/105) |
| Gender |  |  |  |
| Male | 85% (428/502) | 61% (217/354) | 75% (79/105) |
| Female | 15% (74/502) | 39% (137/354) | 25% (26/105) |
| Marital status |  |  |  |
| Single | 66% (325/493) | 54% (194/356) | 52% (55/105) |
| Married | 10% (49/493) | 3% (12/356) | 8% (8/105) |
| Co-habiting | 1% (7/493) | 20% (71/356) | 1% (1/105) |
| Separated | 15% (75/493) | 16% (57/356) | 12% (13/105) |
| Divorced | 4% (20/493) | 5% (17/356) | 6% (6/105) |
| Widowed | 3% (17/493) | 1% (5/356) | 3% (3/105) |
| Long term partner | - | - | 18% (19/105) |
| Children |  |  |  |
| Had children | 47% (231/490) | 69% (243/352) | 52% (54/104) |
| Had children living with them  You or partner currently pregnant | - | 20% (71/355) | 4% (4/104)  3% (3/105) |
| Nationality and political status |  |  |  |
| Irish | 79% (394/502) | 90% (321/356) | 74% (78/105) |
| British | 5% (24/502) | 3% (10/356) | 7% (7/105) |
| European (outside Ireland and UK) | 5% (23/502) | 1% (2/356) | 14% (15/105) |
| Non-European | 12% (62/502) | 1% (2/356) | 5% (5/105) |
| Asylum seeker/refugee | 15% (77/502) | 1% (2/356) | 1% (1/104) |
| Irish traveller Community  Highest level of education  No formal education  Primary  Lower secondary  Upper secondary  Post secondary  Third level | 3% (14/502)  -  -  -  -  - | 5% (18/356)  -  -  -  -  - | 1% (1/104)  11% (11/103)  18% (19/103)  45% (46/103)  17% (17/103)  -  10% (10/103) |
| Accommodation |  |  |  |
| Hostel | 80% (392/489) | 65% (231/356) | 51% (53/104) |
| B & B | 12% (60/489) | 35% (125/356) | 3% (3/104) |
| Sleeping rough | 5% (23/489) | - | 6% (6/104) |
| Staying with friends | 3% (14/489) | - | 9% (9/104) |
| Staying with relatives | combined above | - | 6% (6/104) |
| Owned/renting accommodation | - | - | 26% (27/104) |
| Primary reasons for becoming homeless* |  |  |  |
| Family related problems | 32% (113/355) | 37% (133/348) | 23% (24/103) |
| Financial reasons | 20% (71/355) | 13% (45/348) | 24% (25/103) |
| Evictions | 7% (24/355) | 7% (25/348) | 3% (3/103) |
| Drug/alcohol addiction | 24% (84/355) | 31% (107/348) | 28% (29/103) |
| Mental health | 5% (18/355) | - | 2% (2/103) |
| Release from prison | 3% (11/355) | - | 4% (4/103) |
| Other | 10% (34/355) | - | 16% (16/103) |

*The current study recorded the response to this question in free text and categorised it after the interview. The 1997 and 2005 studies offered categories and therefore the groups may not be directly comparable
