# Supplementary material for: Health and use of health services of people who are homeless and at risk of homelessness who receive free primary health care in Dublin
Source: BMC Health Serv Res. 2015 Feb 12;15:58. doi: 10.1186/s12913-015-0716-4 (PMC4343065; doi:10.1186/s12913-015-0716-4)
Supplement: Additional file 2: Table S2. — Health and morbidity of participants in current study at baseline compared with previous Irish studies. [file 12913_2015_716_MOESM2_ESM.docx]

**Additional file 2: Table S2: Health and morbidity of participants in current study at baseline compared with previous Irish studies**

|  | Holohan et al 1997 (n=502)  %(n) | O’Carroll et al 2005 (n=356)  %(n) | Current study 2011 baseline data (n=105)  %(n) |
| --- | --- | --- | --- |
| Self-rated physical health |  |  |  |
| Good to excellent | 57% (274/484) | 46% (165/355) | 70% (74/105) |
| Fair to poor | 43% (210/484) | 54% (190/355) | 30% (31/105) |
| Mental or physical health condition (limited conditions)* | 78% (372/474) | 84% (298) | 90% (94/105) |
| Average number of physical or mental health conditions*  Mental health condition (ever)  Physical health condition (ever) | 2.8 (range 1-10, n=372)  -  - | -  -  - | 3.3 (range 1-11, n=105)  70% (73/105)  100% (105/105) |
| Receiving prescription medications (self report)  Average number of prescriptions (self report) | 32% (153/474)  - | 49% (173/354)  - | 81% (85/105)  2.2 (range 1-10, n-99) |
| Receiving over-the-counter medications (self report) | - | - | 23% (24/105) |
|  |  |  |  |
| Physical health (if participant ever had condition) |  |  |  |
| Anaemia | - | - | 12.5% (13/104) |
| Diabetes mellitus | 3% (12/474) | 1% (4/346) | 8% (8/105) |
| Hypertension | 13% (60/471) | 14% (49/341) | 22% (23/105) |
| Arthritis | 14% (65/474) | 14% (48/345) | 6% (6/105) |
| Heart disease | 5% (24/474) | 6% (20/339) | 10% (10/104) |
| Deep vein thrombosis (DVT) | - | - | 19% (20/105) |
| Pulmonary embolism (PE) | - | - | 4% (4/105) |
| Heart attack | - | - | 2% (2/105) |
| Angina | - | - | 10% (10/105) |
| Stroke | - | - | 2% (2/105) |
| Epilepsy | 5% (25/474) | 8% (28/346) | 8% (8/105) |
| Tuberculosis | 3% (13/474) | 3% (10/336) | 2% (2/105) |
| Respiratory disease** | 16% (75/474) | 16% (57/348) | 38% (40/105) |
| Asthma  COPD  Chest infection or bronchitis | - | 23% (80/346) | 21% (22/105)  3% (3/105)  31% (32/105) |
| Peptic ulcer disease | 14% (65/474) | 11% (37/346) | 21% (22/105) |
| Other gastro-intestinal tract disorder | - | - | 27% (28/105) |
| Cancer | - | - | 3% (3/105) |
| Kidney problems | - | - | 10% (10/105) |
| Migraine/regular severe headaches | - | - | 31% (32/105) |
| Dental problems*** | 37% (176/474) | 53% (177/337) | 48% (50/105) |
| Skin problems (including skin ulcers, psoriasis, eczema and other skin) | 16% (76/474) | 14% (47/347) | 52% (55/105) |
| Skin ulcers  Psoriasis  Eczema  Joint problems | -  -  -  - | -  -  -  - | 23% (24/105)  3% (3/105)  11% (12/105)  23% (24/105) |
| Back problems | - | - | 17% (18/105) |
| Foot problems | 21% (101/474) | 21% (76/354) | 19% (20/104) |
| Eye problems**** | - | - | 31% (32/105) |
| Sexually transmitted infection | - | - | 8% (8/105) |
|  |  |  |  |
| Blood-borne diseases |  |  |  |
| HIV positive | - | 6% (22/345) | 6% (6/105) |
| AIDS | - | - | 0% (0/105) |
| Hepatitis B | - | 5% (18/338) | 3% (3/104) |
| Hepatitis C | - | 36% (124/343) | 23% (24/104) |
|  |  |  |  |
| Mental health |  |  |  |
| Self-rated mental health |  |  |  |
| Good to excellent | - | - | 75% (79/105) |
| Fair to poor | - | - | 25% (26/105) |
| Depression (self-report)  Depression based on PHQ-9 score 10 or more | 33% (154/474) | 51% (174/343) | 50% (52/105)  41% (43/105) |
| PHQ-9 score 0-4: no depression | - | - | 24% (25/105) |
| PHQ-9 score 5-9: mild depression | - | - | 35% (37/105) |
| PHQ-9 score 10-14:moderate depression | - | - | 21% (22/105) |
| PHQ-9 score 15-19: moderately severe | - | - | 10% (10/105) |
| PHQ-9 score 20-27: severe depression | - | - | 10% (11/105) |
| Anxiety (self-report)  Anxiety based on a GAD-7 score of 10 or greater | 28% (131/474) | 42% (143/339) | 36% (38/105)  47% (49/105) |
| GAD-7 score 0-4: no anxiety | - | - | 28% (29/105) |
| GAD-7 score 5-9: mild anxiety | - | - | 26% (27/105) |
| GAD-7 score 10-14: moderate anxiety | - | - | 30% (31/105) |
| GAD-7 score 15-21: severe anxiety | - | - | 17% (18/105) |
| Bipolar disorder | - | - | 6% (6/105) |
| Addiction | - | - | 39% (41/105) |
| Schizophrenia (self-report) | - | 11% (36/339) | 13% (14/105) |
| Other mental health disorder | - | - | 13% (13/104) |
| Suicidal feelings in last 6 months |  |  | 18% (19/105) |
| Suicide attempt in last 6 months |  |  | 9% (9/105) |
|  |  |  |  |
| Quality of life |  |  |  |
| Self-rated quality of life |  |  |  |
| Good to excellent | - | - | 72% (76/105) |
| Fair to poor | - | - | 28% (29/105) |
| SF-12 score  PCS - Physical component summary (mean± SD, range)  MCS – Mental component summary (mean± SD, range) |  |  | 48.1±10.8, 14.0-70.0  43.5±12.5, 6.7-66.2 |

*****Our list of mental and physical health problems was much more extensive than previous studies. For comparison this has been limited to diabetes mellitus, hypertension, arthritis, heart disease, epilepsy, tuberculosis, respiratory disease, peptic ulcer disease, depression, anxiety, dental problems, skin problems, foot problems, Hepatitis C, HIV and Hepatitis B

** Respiratory disease- this variable was created by combining asthma, COPD, chest infection, PE and TB

***Dental problems includes missing teeth, toothache, caries and dentures

**** Eye problems includes requiring glasses and loss of sight due to trauma
