# Supplementary material for: Health and use of health services of people who are homeless and at risk of homelessness who receive free primary health care in Dublin
Source: BMC Health Serv Res. 2015 Feb 12;15:58. doi: 10.1186/s12913-015-0716-4 (PMC4343065; doi:10.1186/s12913-015-0716-4)
Supplement: Additional file 3: Table S3. — Health behaviours of participants in current study at baseline compared with previous Irish studies. [file 12913_2015_716_MOESM3_ESM.docx]

**Additional file 3: Table S3: Health behaviours of participants in current study at baseline compared with previous Irish studies**

|  | | Holohan et al 1997 (n=502)  %(n) | | O’Carroll et al 2005 (n=356)  %(n) | | Current study 2011 baseline data (n=105)  %(n) | |
| --- | --- | --- | --- | --- | --- | --- | --- |
| Smoking |  | |  | |  | |  |
| Smoking (current) | 78% (373/480) | | 90% (321) | | 82% (86/105) | |  |
| Smoking past user  >30 cigarettes a day or 3oz per week | 6% (30/480)  32% (120/370) | | - | | 9% (9/105)  13% (11/86) | |  |
|  |  | |  | |  | |  |
| Alcohol |  | |  | |  | |  |
| Alcohol use (current or past) | - | | - | | 90% (95/105) | |  |
| Alcohol use current | 53% (266/502) | | - | | 58% (61/105) | |  |
| Alcohol use past | - | | - | | 32% (34/105) | |  |
| AUDIT-C problem drinking  Problem drinking (>14 units* for women or 21 units for men) | -  25% (127/502) | | -  - | | 53% (56/105)  26% (27/105) | |  |
|  |  | |  | |  | |  |
|  |  | |  | |  | |  |
| Drug use (current or past) | 29% (136/470) | | 64% (228) | | 60% (63/105) | |  |
| Drug use current user | - | | 23% (83/354) | | 33% (35/105) | |  |
| Drug use past user | - | | 42% (147/354) | | 27% (28/105) | |  |
| Accidental overdose - in last 3 months |  | |  | | 3% (3/105) | |  |
|  |  | |  | |  | |  |
| Injecting behaviours |  | |  | |  | |  |
| Injected drugs – ever | - | | 48% (168/348) | | 44% (46/105) | |  |
| Injected with a needle/syringe used by  someone else – ever | - | | - | | 39% (18/46) | |  |
| Re-used own needle/syringe – ever | - | | - | | 56% (26/46) | |  |
| Used a filter/spoon/flush used by  someone else – ever | - | | - | | 43% (20/46) | |  |
| Injected drugs in last three months | - | | - | | 22% (23/105) | |  |
|  |  | |  | |  | |  |
| Sex and condom use |  | |  | |  | |  |
| Sex with regular partner in last 3 months | - | | - | | 33% (35/105) | |  |
| Condoms used on every occasion | - | | - | | 23% (8/35) | |  |
| Sex with someone other than regular  partner in last three months | - | | - | | 10% (10/103) | |  |
| Condoms used on every occasion | - | | - | | 40% (4/10) | |  |
| Ever paid for sex | - | | - | | 20% (21/104) | |  |
| Condoms used on every occasion | - | | - | | 71% (15/21) | |  |
| Ever sold sex | - | | - | | 0% (0/104) | |  |

*For this study units per week were based on units per typical drinking day X frequency of drinking. For previous studies, units per week were based on units drank on an average day X 7.
