# Supplementary material for: Health and use of health services of people who are homeless and at risk of homelessness who receive free primary health care in Dublin
Source: BMC Health Serv Res. 2015 Feb 12;15:58. doi: 10.1186/s12913-015-0716-4 (PMC4343065; doi:10.1186/s12913-015-0716-4)
Supplement: Additional file 4: Table S4. — Medications information. [file 12913_2015_716_MOESM4_ESM.docx]

**Additional file 4: Table S4: Medications information**

|  | | From self report, current medication n=105 | | From patient records in previous 6 months  Baseline, n=66 | | From patient records in previous 6 months  12 month follow-up, n=74 |
| --- | --- | --- | --- | --- | --- | --- |
| Prevalence of prescribing | | 81% (85/105) | | 64% (42/66) | | 43% (32/74) |
| Average number of medications prescribed (±SD, range) | | 2.15 (±2.1, 0-10) | | 6.3 (±8.5, 0-38) | | 6.9 (±15.3, 0-97) |
| 1 medication prescribed | | 29% (25/85) | | 12% (5/42) | | 16% (5/32) |
| 2 medications | | 26% (22/85) | | 12% (5/42) | | 16% (5/32) |
| 3 medications  4+ medications | | 14% (12/85)  24% (20/85) | | 2% (1/42)  74% (31/42) | | 13% (4/32)  56% (18/32) |
|  |  | |  | |  | |
