# Supplementary material for: Health and use of health services of people who are homeless and at risk of homelessness who receive free primary health care in Dublin
Source: BMC Health Serv Res. 2015 Feb 12;15:58. doi: 10.1186/s12913-015-0716-4 (PMC4343065; doi:10.1186/s12913-015-0716-4)
Supplement: Additional file 5: Table S5. — Use of health services by participants in current study compared with previous Irish studies. [file 12913_2015_716_MOESM5_ESM.docx]

**Additional file 5: Table S5: Use of health services by participants in current study compared with previous Irish studies**

|  | Holohan et al 1997 (n=502)  %(n) | O’Carroll et al 2005 (n=356)  %(n) | Current study 2011 baseline data (n=105)  %(n) |
| --- | --- | --- | --- |

| Current medical card | | | 55% (257/467) | | 55% (196/354) | | 59% (62/105) |
| --- | --- | --- | --- | --- | --- | --- | --- |
| Reasons for no medical card: No stable address  Applied, waiting for response  Don’t know how to get one  Problems with form  Difficulty in getting a GP  Other reason | | | -  -  -  -  -  - | | -  -  -  -  25%  - | | 7% (3/41)  44% (18/41)  5% (2/41)  15% (6/41)  0  29% (12/41) |
| Rating health services for homeless  Very good to excellent  Good  Fair to poor  Use of medical services in last 6 months | | | -  -  - | | -  -  - | | 61% (63/103)  29% (29/103)  11% (11/103) |
| Visiting GP  Average number of times visiting GP (self report) | | | 52% (262/502)  5.1 (range 1-48, n=262)^*^ | | 62% (221/356) | | 82% (86/105)  6.5 (range = 1-30, n=81) |
| Nurse in Safetynet clinic (Granby & MQI) | | | - | |  | | 44% (46/105) |
| Attended A&E  Average number of times visiting A & E | | | 22% (112/502)  1.7 (range 1-20, n=112) | | 37% (128/346) | | 29% (30/105)  2.0 (range 1-10, n=30) |
| Attended OPD | | | 20% (102/502) | | 27% (93/347) | | 17% (18/105) |
| Hospital inpatient | | | - | | 19% (68/356) | | 22% (23/105) |
| Public health nurse | | | 1% (39/502) | | - | | 9% (9/105) |
| Nurse in any other hostel  Dentist  Optician  Social worker  Community welfare officer | | | -  -  -  17% (85/502)  41% (208/502) | | -  -  -  -  - | | 3% (3/105)  16% (17/105)  12% (13/105)  24% (25/105)  27% (28/105) |
|  | | |  | |  | |  |
| Use of mental health services in last 6 months |  |  | |  | |  |  |
| One to one counselling/therapy | | | - | | - | | 23% (24/104) |
| Group counselling/therapy | | | - | | - | | 4% (4/104) |
| Alcoholics anonymous | | | - | | - | | 6% (6/104) |
| Narcotics anonymous | | | - | | - | | 11% (11/104) |
| Residential drug treatment | | | - | | - | | 2% (2/104) |
| Supervised detox (outpatient) | | | - | | - | | 1% (1/104) |
| Psychiatric inpatient unit | | | - | | - | | 6% (6/104) |
| Psychiatric outpatient unit | | | 15% (76/502) | | - | | 17% (18/104) |
| Community psychiatric nurse | | | 13% (65/502) | | - | | 2% (2/104) |
| Addiction psychiatry | | | - | | - | | 1% (1/104) |
| Phoneline service (e.g. Samaritans) | | | - | | - | | 1% (1/104) |
| On waiting list for any of these services | | | - | | - | | 3% (3/104) |
|  | | |  | |  | |  |
| Use of drug services | | |  | |  | |  |
| Methadone (past or present) | | | - | | - | | 35% (37/105) |
| Methadone current use | | | - | | - | | 29% (30/105) |
| On waiting list for methadone treatment | | | - | | - | | 4% (4/105) |
| Needle exchange (in last 6 months) | | | - | | - | | 21% (22/104) |
|  | | |  | |  | |  |
| Other service | | |  | |  | |  |
| Day care centre | | | - | | - | | 38% (39/104) |
| Social worker | | | - | | - | | 24% (25/105) |
| Community welfare officer | | | - | | - | | 26% (28/105) |
| Dentist | | | - | | - | | 16% (17/105) |
| Optician | | | - | | - | | 12% (13/105) |
|  | | |  | |  | |  |
| Vaccinations | | |  | |  | |  |
| Flu vaccine in last year | | | - | | - | | 56% (58/103) |
| Hepatitis B (at least one vaccine) | | | - | | - | | 33% (34/103) |
| Hepatitis B (all three vaccines) | | | - | | - | | 17% (17/103) |
| Hepatitis B blood test (post-vaccine) | | | - | | - | | 88% (15/17) |
|  | | |  | |  | |  |
| Blood tests (ever received) | | |  | |  | |  |
| Hepatitis B | | | - | | - | | 57% (59/103) |
| Hepatitis C | | | - | | - | | 57% (59/103) |
| HIV | | | - | | - | | 56% (58/103) |
| AIDS | | | - | | - | | 56% (58/103) |
| Tuberculosis | | | - | | - | | 37% (38/103) |
